# Warts on hand: a rare clinical image

**DOI:** 10.11604/pamj.2022.41.343.34821

**Published:** 2022-04-28

**Authors:** Mayur Bhaskar Wanjari, Tejaswee Lohakare

**Affiliations:** 1 Department of Research and Development, Jawaharlal Nehru Medical College, Datta Meghe Institute of Medical Sciences, Sawangi, Wardha, Maharashtra, India,; 2 Department of Child Health Nursing, Smt. Radhikabai Meghe Memorial College of Nursing, Datta Meghe Institute of Medical Sciences, Sawangi, Wardha, Maharashtra, India

**Keywords:** Warts, papillomavirus, contagious

## Image in medicine

Warts are a type of skin infection caused by the human papillomavirus (HPV). The disease causes rough, skin-coloured bumps to form on the skin. The virus is contagious. Warts can be spread by touching someone who has them. We are presenting a case of 21 years old female who comes to the skin outpatient department with the complaint of skin-coloured bumps on the middle finger. The physician inspected the patient hand and suggested a skin biopsy of the infected area. A biopsy sample was taken for the skin growth test for the HPV, and it revealed the patient had an infection of HPV. After all examinations, the patient was diagnosed with warts and given medical management on that.

**Figure 1:**
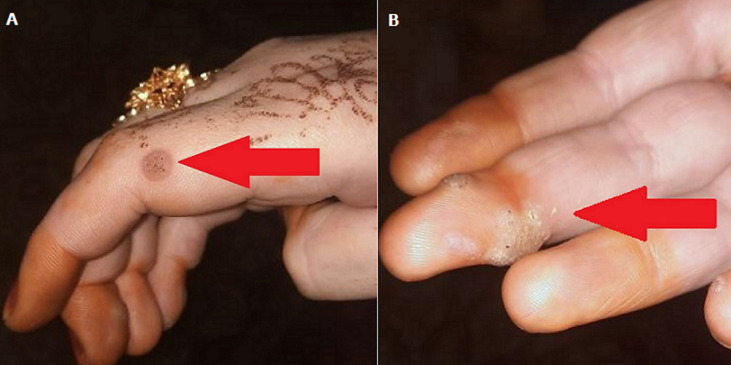
right-hand skin-coloured bumps (A,B)

